# Autism spectrum disorder and a possible role of anti-inflammatory treatments: experience in the pediatric allergy/immunology clinic

**DOI:** 10.3389/fpsyt.2024.1333717

**Published:** 2024-06-24

**Authors:** Harumi Jyonouchi

**Affiliations:** ^1^ Department of Pediatrics, Saint Peter’s University Hospital, New Brunswick, NJ, United States; ^2^ Department of Pediatrics, Rutgers University-Robert Wood Johnson School of Medicine, New Brunswick, NJ, United States

**Keywords:** ASD (autism spectrum disorder), biologics, immunomodulating agents, neuroinflammation, COVID-19 (coronavirus disease 2019)

## Abstract

Autism spectrum disorder (ASD^1^) is a behaviorally defined syndrome encompassing a markedly heterogeneous patient population. Many ASD subjects fail to respond to the 1^st^ line behavioral and pharmacological interventions, leaving parents to seek out other treatment options. Evidence supports that neuroinflammation plays a role in ASD pathogenesis. However, the underlying mechanisms likely vary for each ASD patient, influenced by genetic, epigenetic, and environmental factors. Although anti-inflammatory treatment measures, mainly based on metabolic changes and oxidative stress, have provided promising results in some ASD subjects, the use of such measures requires the careful selection of ASD subjects based on clinical and laboratory findings. Recent progress in neuroscience and molecular immunology has made it possible to allow re-purposing of currently available anti-inflammatory medications, used for autoimmune and other chronic inflammatory conditions, as treatment options for ASD subjects. On the other hand, emerging anti-inflammatory medications, including biologic and gate-keeper blockers, exert powerful anti-inflammatory effects on specific mediators or signaling pathways. It will require both a keen understanding of the mechanisms of action of such agents and the careful selection of ASD patients suitable for each treatment. This review will attempt to summarize the use of anti-inflammatory agents already used in targeting ASD patients, and then emerging anti-inflammatory measures applicable for ASD subjects based on scientific rationale and clinical trial data, if available. In our experience, some ASD patients were treated under diagnoses of autoimmune/autoinflammatory conditions and/or post-infectious neuroinflammation. However, there are little clinical trial data specifically for ASD subjects. Therefore, these emerging immunomodulating agents for potential use for ASD subjects will be discussed based on preclinical data, case reports, or data generated in patients with other medical conditions. This review will hopefully highlight the expanding scope of immunomodulating agents for treating neuroinflammation in ASD subjects.

## 1 Introduction

ASD is a complex developmental disorder, mostly defined by behavioral symptoms and its onset and progress is likely to be affected by multiple genetic and environmental factors (1). Such genetic and environmental factors likely vary in ASD subjects, resulting in markedly heterogeneous patients that all fall under the current diagnostic criteria of ASD. This makes it difficult to treat ASD subjects with ‘one size fits all’ measures. It would be ideal if tailor-made approaches based on each ASD subject’s genetic/epigenetic/environmental conditions could be created. Instead, the 1^st^ line treatment measures for ASD are behavioral and pharmacological interventions. However, these measures are not universally effective. Primary care providers may be consulted by frustrated parents regarding other treatment options which are often promoted by practitioners of complementary and alternative medicine (CAM). However, such CAM measures are often not based on sound scientific rationale and rigorous clinical trials. In contrast, treatment measures targeting specific molecules or pathways of neuroinflammation may provide alternative treatment options for some ASD subjects who are found to have evidence of neuroinflammation associated with specific mechanisms. This review will discuss anti-inflammatory measures that have been tried or can be applied to ASD subjects based on scientific rationale.

Inflammation has long been indicated in the pathogenesis of ASD through multiple lines of evidence. Epidemiological studies have indicated that maternal inflammation caused by infectious and non-infectious triggers during pregnancy are associated with an increased risk of ASD (2, 3). As direct evidence of neuroinflammation, neuroglial activation in the presence of inflammatory mediators has been shown in the brain of ASD subjects (4). Further analysis revealed that maternal inflammation occurring in the 1^st^ and 2^nd^ trimesters has a role in developmental impairment of offspring, irrespective of triggering events (5, 6). Such findings led to the creation of one of the most rigorously studied animal models of autism, maternal immune activation (MIA). In this rodent model, maternal sterile inflammation is induced by injection of endotoxin during the 2^nd^ trimester, and this leads to ASD like developmental symptoms in offspring later in life (1, 7). Such prolonged effects of maternal inflammation not associated with specific pathogens is partly explained by the reprogramming of innate immune responses. That is, epigenetic changes following potent immune stimuli result in persistent changes in innate immunity, referred as to innate immune memory (IIM) (8–10) MIA may cause inflammation skewed IIM, referred as to trained immunity (TI) (8, 10). In fact, mal-adapted TI is implicated in the pathogenesis of numbers of chronic neuropsychiatric conditions (8, 9). It has also been shown that maternal derived interleukin-6 (IL-6) plays a key intermediary in the MIA model (11). Further study revealed the importance of placental IL-6 for the development of the fetal brain and subsequent behavioral changes (12). These results indicate that maternal sterile inflammation can cause profound and lasting effects on offspring.

Apart from MIA, cognitive development is known to be affected by genetically altered immune responses prone to neuroinflammation. Gene variants associated with increased risk of ASD often cause aberrant immune responses and subsequent inflammatory condition (1). For example, variants of tuberous sclerosis complex 1 and 2 (TSC1/TSC2) are associated with inflammatory conditions caused by aberrant activation of the mTOR (mammalian target of rapamycin) pathway (13). ASD subjects are also characterized by a high frequency of comorbid inflammatory conditions such as chronic GI inflammation, which may also be indicative of inflammation prone immune conditions (14–17).

If neuroinflammation does play a role in the pathogenesis of ASD, questions may arise as to whether there is direct evidence of neuroinflammation in the brain of these individuals, and if so, what type of cells are contributing to neuroinflammation. Microglial cells are one of the key innate immune cells in the brain, and they are resident macrophages in the CNS. They are grossly classified as either proinflammatory (M1) or anti-inflammatory alternatively activated (M2) microglial cells, although they exhibit diverse phenotypes (18). Embryogenic microglial cells, along with astrocytes, are thought to play a crucial role in brain development, regulating neurogenesis, neuronal migration, and synaptic plasticity (19–21). Microglial cells also act as major innate immune cells in the CNS throughout life, serving as a sensor of the CNS microenvironment, and they are easily activated by various stimuli (20). Such activated microglial cells play a pivotal role in controlling infection, inflammation, and injury in the CNS (19, 20). However, dysregulated activation of microglial cells can cause chronic neuroinflammation. Bone marrow (BM) derived macrophages can also infiltrate to the CNS under conditions when the blood brain barrier (BBB) is impaired, and can transform into BM derived microglial cells, exerting proinflammatory actions (22). For example, a pathogenic role of microglial cells is illustrated in Rett syndrome. *MECP2* pathogenic variants cause Rett syndrome which is characterized by progressive developmental disorder and ASD behavioral symptoms. It has been shown that loss of function (LOF) *MECP2* pathogenic variants cause dysregulated inflammatory responses of microglial cells, partly due to both an increase in mitochondrial production of reactive oxygen species (ROS) and a decrease in ATP production (19, 23–25).

It is also of note that conditions affecting other organs can exert significant effects on CNS inflammation in ASD patients. This may be partly explained by the effects of BM derived microglial cells (22), circulating mediators released by the affected organs, and stimuli through the sensory nervous system. In fact, microglial cells in the CNS are known to be affected by stimuli derived from other organs (19–21). MIA induced developmental impairment are in part attributed to the effects of maternally derived inflammatory mediators that affect fetal brain cells, especially microglial cells (1). The gut microbiome is also likely to affect brain development through various mechanisms and its role in the pathogenesis of neurodegenerative disease has been a focus of intense research (26).

The evidence that supports a role for neuroinflammation mediated by innate immune cells in the brain, at least in some ASD patients, are summarized as described above. However, in each ASD individual, the mechanisms of neuroinflammation may vary depending on various genetic, epigenetic, and environmental factors that affect development of the brain and the immune system. Understanding the dynamic mechanisms of neuroinflammation will help us apply anti-inflammatory measures as treatment options for ASD patients. In the following section, anti-inflammatory measures used for targeting ASD subjects, based on scientific rationale and trial data, will be discussed first. Then, possible application of other immunomodulating agents for treating ASD subjects will be discussed. This will include emerging agents developed recently for treating autoimmune/autoinflammatory and other chronic inflammatory conditions. For these agents, secondary to scant data of clinical trials in ASD subjects, discussion will be based on pre-clinical data, case reports, results generated from use in other medical conditions.

## 2 Metabolic factors associated with neuroinflammation in ASD and treatment measures targeting metabolic changes or factors causing such changes

### 2.1 Lipid metabolites and their signaling pathways

1) *COX1 and COX2*: Lipids are major components of the brain and lipid metabolites act as regulatory molecules for both brain development and homeostasis. Major lipid metabolites that serve as lipid mediators are prostaglandins (PGs) and leukotrienes (LTs) metabolized by arachidonic acid (AA) and other unsaturated fatty acids by cyclooxygenases (COXs) and lipoxygenases (LOXs), respectively (27). PGE2 signaling has been known to play a role in brain morphogenesis (28, 29) and impaired COX2/PEG2 signaling has been associated with ASD pathogenesis in the MIA model (29, 30). The COX pathway involves two rate limiting enzymes, COX-1 and COX-2. COX-1 is expressed constitutively in all the cells, while COX-2 is induced by inflammatory mediators and expressed mainly in the CNS, kidney, thymus, and gastrointestinal (GI) tract (31). Both endotoxin which is used for inducing MIA, and inflammatory mediators generated in the MIA model (interleukin-1ß (IL-1ß), IL-6, tumor necrosis factor-α (TNF-α), type 1interferons (IFNs), and AA), induce COX-2 (31, 32). In addition, evidence suggests that COX-2 mediates N-methyl-D aspartate (NMDA) neurotoxicity (33).
2) *COX2 and ASD*: In the previous studies that used peripheral blood monocytes from ASD subjects, as surrogates of microglial cells, increased production of the above-described cytokines in response to innate immune stimuli were observed (34). Such increases occur in ASD subjects that have a history of fluctuating behavioral symptoms and cognitive functioning following microbial infection (34). Interestingly, COX-2 and PGE2 were reported to be elevated in plasma of ASD subjects along with lower levels of α-synuclein (35). Therefore, blocking COX-2 may have the potential to attenuate neuroinflammation and subsequent neuronal damage in ASD subjects. On the other hand, up-regulation of COX-2 in the brain may be neuroprotective, partly regulating blood flow in the brain (31). It is of note that major adverse reactions associated with COX-2 inhibitors are cardiovascular events (36).

Clinical trial data of COX-2 inhibitors in ASD subjects has been scant. Only one randomized, double-blind, placebo-controlled trial addressed the efficacy of celecoxib, a COX-2 inhibitor, on behavioral symptoms in ASD subjects when celecoxib was given as an adjuvant treatment for risperidone administered for 10 weeks; behavioral symptoms were evaluated with the use of the aberrant behavior checklist (ABC) (37). The authors report statistically significant improvement in ABC subscales of irritability, lethargy, and stereotypy (37). In the author’s clinic, attenuation of behavioral symptoms by celecoxib, a COX2 inhibitor, were also observed frequently when celecoxib was administered for controlling exacerbation of ASD behavioral symptoms, following viral syndromes, like influenza. Pioglitazone exerts various anti-inflammatory effects including suppression of COX-2 expression on microglial cells (38). Beneficial effects of pioglitazone have been reported in traumatic brain injury (38). In one study, pioglitazone is also reported to have attenuated ASD behavioral symptoms (39).

In summary, COX-2 inhibitors may be beneficial for ASD subjects with evidence of COX2 activation, especially in the acute and/or subacute stages. However, a caution is necessary for its long-term use.

### 2.2 Tryptophan metabolism

1) *Kynurenine pathway*: Tryptophan is an essential amino acid and a precursor of the metabolites that affect the functioning of multiple organs, including the brain. In the immune system, most tryptophan is mainly metabolized in the kynurenine pathway (40, 41). Multiple cytokines generated by innate immune responses including type 1 IFNs, IL-1ß, and TNF-α, activate rate-limiting enzymes of indoleamine 2,3-dioxygenases (IDO1/IDO2) in the kynurenine pathway (40). Most cells including immune cells express IDO1 and this enzyme often remains up-regulated in chronic inflammation. Tryptophan depletion caused by IDO activation and accumulation of kynurenine pathway metabolites result in immunosuppression, partly through facilitating induction of regulatory T (Treg) cells (40–43). However, metabolites of the kynurenine pathway can also cause toxic effects on the brain and such toxic effects are implicated in the pathogenesis of neuropsychiatric conditions such as schizophrenia (42). Therefore, in the acute and subacute stages of neuroinflammation, modulating IDO activity may provide protection against neuroinflammation.
2) *Serotonin pathway*: Another pathway of tryptophan metabolism is the serotonin pathway, in which tryptophan is metabolized to serotonin (5-hydroxytryptophan, 5-HT). 5-HT is mainly produced by enterochromaffin cells in the gut, but it is also produced in the brain (40, 42). 5-HT is then metabolized to melatonin, a circadian hormone regulating sleep (42). Serotonin produced in the gut conveys signals to intestinal neurons, affecting various functions of the GI tract (42). 5-HT production in the gut is greatly affected by the gut microbiome (44). Both kynurenine and 5-HT pathways compete each other, and subtle changes in tryptophan metabolism are expected to affect the functions of multiple organs.
3) *Potential effects of maladapted IIM and Tryptophan metabolism on neuroinflammation*: Tryptophan metabolized by IDO1/2 will be metabolized to kynurenic acid (KYNA), which is thought to be neuroprotective, by exerting antagonistic effects on NMDA. On the other hand, quinolinic acid (QUIN), a down-stream metabolite of KYNA, activates NMDA receptors (41, 45). QUIN produced by microglial cells, is implicated in the pathogenesis of neuropsychiatric diseases such as depression (45, 46). In contrast, in the disorders of dopaminergic transmission like schizophrenia, excessive KYNA may be harmful, causing down-regulation of NMDAR signaling (45). In addition, general immunosuppressive actions exerted by activation of the kynurenine pathway may cause chronic immunosuppression through facilitating differentiation of Treg cells (42). Excessive actions of Treg cells make subjects vulnerable to recurrent infection and more susceptible to malignancy.

Changes in kynurenine pathway metabolites have also been reported in those diagnosed with ASD. For example, increased urinary concentrations of neurotoxic tryptophan metabolites were reported in ASD subjects (47). Polymorphism of NMDAR subunits, target molecules of kynurenine metabolites, have also been reported in subjects with ASD (48), along with altered levels of other tryptophan metabolites (49, 50).

High circulating levels of 5-HT, which essentially reflects 5-HT produced in the gut and stored in platelets, have been reported in about one third of ASD subjects (51). Changes in 5-HT levels may be associated with changes in gut serotonin metabolism and/or changes in clearance of 5-HT from both the liver and the lung (52). However, associations between hyperserotonemia and characteristic ASD behavioral symptoms have not been consistently shown (52). Likewise, selective serotonin reuptake inhibitors (SSRIs) that inhibit the actions of the serotonin reuptake transporter (SERT) do not exert universally beneficial effects on those diagnosed with ASD. These findings indicate that there are complex underlying mechanisms at play. Interestingly, an analysis of principal pathogenetic components and biological endophenotypes including serotonin blood levels, identified associations with immune dysfunction in ASD subjects (53). In the same study population, the immune component provided the largest contributions to phenotypic variance (54); these results support the effects of the immune activation on serotonin metabolism in ASD subjects. Notable findings from studies addressing changes in serotonin metabolites in ASD subjects at molecular levels are summarized below:

#### 2.2.1 SERT polymorphism

Since tryptophan is a highly charged molecule, it requires SERT, an active transporter, that brings 5-HT through cell membranes and the transported 5-HT is then inactivated by monoamine oxidase (18). The modulation of actions of SERT impacts the development of the centric and enteric nervous systems (52, 55). The gene Slc6a4, which encodes SERT, has been the focus of intensive research, examining its association with the pathogenesis of common neuropsychiatric conditions. A finding of a gain of function (GOF) pathogenic variant of the SERT gene led to the development of an animal model of ASD, ‘SERT Ala56 mouse’ (56). In this murine ASD model, the GOF SERT gene mutation causes excessive activation of p38MAPK (57), and resultant hyper-clearance of 5-HT and hyper-sensitivity to 5-HT receptors in the nervous system (56). The GOF SERT mutation causes hyperserotonemia as was observed in SERT knockout mice (55, 56). Altered levels of 5-HT in both the CNS and gut, have also been reported in the MIA, a rodent model of autism (58). In ASD subjects with frequent activation of innate immunity through immune insults, changes in tryptophan metabolism are likely to occur, affecting or exacerbating pre-existing neuropsychiatric symptoms. This is partly because IL-1β and other inflammatory cytokines generated by innate immune responses activate both IDO1 and SERT (41, 42, 56, 57).

#### 2.2.2 Effects of minocycline on tryptophan metabolism

One of the immunomodulating agents that can affect the kynurenine pathway is minocycline. This second-generation tetracycline antibiotic exerts anti-inflammatory and immunoregulatory actions partly through suppressing cytokine production and microglial cell activation. Minocycline’s unique neuroprotective effects have been partly attributed to its direct suppression of IDO1 in the presence of retinoic acid (vitamin A): IDO inhibition by minocycline results in reduced productions of neurotoxic tryptophan metabolites (59). The neuroprotective effects of minocycline are also thought to be associated with its inhibitory effects on GSK3ß (60). Neuroprotective effects of minocycline are best demonstrated in neuroinflammation caused by reperfusion injury (61). In the animal model of white matter disease induced by intracerebral hemorrhage, protective effects of minocycline are attributed to its suppressive action on MAPK signaling mediated by TGF-ß (62). Neuroprotective effects of minocycline has also been shown to improve stress-induced behavioral changes (60).

Although the effects of minocycline appear promising in animal models and in other medical conditions, the therapeutic effects of minocycline on ASD have not been consistently shown. Recent meta-analysis of minocycline as treatment for neuropsychiatric conditions revealed beneficial effects of minocycline on schizophrenia, favoring its use as an adjunctive treatment (63). As for ASD, only one randomized, double-blind, placebo-controlled trial of minocycline evaluated its effects as an adjunctive treatment to risperidone (64). In this study, 46 medication-naïve ASD children were treated for 10 weeks with risperidone plus placebo or risperidone plus minocycline (N=23 in each group). Authors reported significant improvement in scores of ABC hyperactivity and irritability subscales, but no effect on inappropriate speech, lethargy, and stereotypy ABC subscales (64).

The above-described findings on the use of minocycline may be partly attributed to the complex effects that multiple genetic and environmental factors have on tryptophan metabolism. It is also possible that prolonged use of minocycline may block the immunosuppressive effects of tryptophan metabolites of KYNA and Treg cells, subsequently increasing the risk of autoimmune conditions. Minocycline may be effective for specific conditions when intricate homeostasis of tryptophan metabolites is impaired, resulting in worsening toxic effects of kynurenine metabolites. Careful selection of ASD subjects will be required for applying minocycline as a treatment option for ASD subjects.

### 2.3 PI3K/Akt/mTOR signaling pathway

Pathogenic variants of multiple genes are known to impose a significant risk for developing ASD and are often associated with regulation of the signaling pathways. Hyperactivation of PI3K (phosphatidylinositol 3 kinase)/Akt (protein kinase B)/mTOR pathway is one of such pathways affected by ASD pathogenic variants (65). This pathway has been shown to exert a crucial role in brain development including corticogenesis and synaptogenesis (65).

1) *PI3K/Akt/mTOR pathway*: mTOR is expressed ubiquitously in all cells as two types of mTOR complexes (mTORC1 and mTORC2) and it plays a major role in cell proliferation and activation. Activation of mTORC1 leads to inhibition of autophagy and Treg cell differentiation (65, 66). mTORC1 responds to signals from nutrients, metabolites and growth factors and it controls cellular functions including energy metabolism and autophagy (66). mTORC2 is positively regulated by the TSC1/2 (tuberous sclerosis complex 1 and 2) and up-stream PI3K signaling. mTORC1 is positively regulated by Akt, and the Akt phosphorylation by mTORC2 result in activation of mTORC1. Such complex interactions are essential for brain morphogenesis, synaptic plasticity, and neuronal regeneration. Therefore, pathogenic variants of genes coding for proteins associated with the PI3K/Akt/mTOR pathway cause various neuropsychiatric and neurodegenerative conditions (66). In addition, activation of this pathway is implicated in the pathogenesis of epilepsy, as typically seen in patients diagnosed with tuberous sclerosis (TS) (67).
2) *ASD phenotype and mTOR signaling pathway*: Previous genetic studies for detecting candidate genes associated with ASD risk have identified several genes that are associated with PI-3K/Akt/mTOR signaling pathway; these include FMR1, PTEN, TSC1, and TSC2 (68). This is not surprising, since patients with pathogenic variants of these genes exhibit ASD phenotypes at high frequency (65). Additional evidence of the role of the PI-3K/Akt/mTOR pathway in ASD pathogenesis has emerged in the murine model of ASD generated by fetal exposure to valproic acid. In this model, multiple mechanisms appear to be involved for the development of the ASD phenotype (69). Involvement of the PI-3K/Akt/mTOR pathway is supported by the finding of improved behavioral symptoms with post-natal administration of rapamycin, an mTOR blocker that predominantly blocks mTORC1 (70). This protective action of rapamycin is partly attributed to prevention of impaired autophagy caused by the over-activation of the PI-3K/Akt/mTOR signaling (70). Others also reported a possible link between the increase in plasma levels of CCL5 (chemokine C-C motif ligand 5) in ASD subjects and activation of the mTOR signaling pathway (71).

The above-described findings may indicate that it is feasible to apply mTOR inhibitors in controlling ASD symptoms. However, mTOR inhibitors exert significant immunosuppressive actions and may not be readily applicable to general ASD population. Current available reports focus on its use in controlling seizure activity and other neurological manifestation in patients who are identified to carry pathogenic variants of TSC and PTEN (67, 72–74). The author reported one ASD case with treatment-resistant seizures for whom sirolimus (rapamycin) was successfully used as an adjunctive treatment to IVIg and anakinra for seizure control (75). In summary, mTOR inhibitors likely have a role in a subset of ASD subjects with identified genetic variants associated with PI-3K/Akt/mTOR signaling pathway and those with specific conditions such as treatment-resistant seizures.

It is also of note, that activation of the PI-3K/Akt/mTOR signaling pathway will impair differentiation of Treg cells as well as autophagy. In such conditions, agents that help recovering autophagic deficiency will provide therapeutic effects. One of such agents is N-acetylcysteine (NAC). NAC has been used as an inexpensive dietary supplements for treating glutathione deficiency associated with multiple medical conditions, by improving glutamatergic neurotransmission through glutathione synthesis (76). In addition, NAC is reported to improve autism like behavior by recovering autophagic deficiency and decreasing Notch-1/Hes-1 pathway activity in rodent models (77). Potential therapeutic effects on NAC on ASD behavioral symptoms (irritability and hyperactivity) have been shown by the recent meta-analysis of randomized controlled trials (8–12 weeks trials) (78) Therefore, NAC may be safely tried in ASD patients with evidence of activation of PI-3K/Akt/mTOR signaling pathway as an adjunct treatment.

### 2.4 Microbiome

In the previous sections, we have discussed the possible roles played by lipid metabolites, tryptophan metabolites, and mTOR signaling activation, in association with neuroinflammation observed in ASD. One factor that may affect all the above-described metabolisms and signaling pathways is the microbiome. Bidirectional communications between the CNS and gut microbiota, often referred as to gut-brain axis, have been studied extensively. Evidence supports a role for the microbiota in the pathogenesis of common neurodevelopmental disorders, including ASD (26). A role of microbiome in neurodevelopmental conditions has been reviewed by many others (79–82). Known key roles that microbiome plays in neuronal development in association with ASD pathogenesis are summarized below:

1) *The role of microbiota in brain development*: The role of microbiota in brain development has been extensively studied in the germ-free mice. Evidence supports its role in multiple neurodevelopmental processes that include maturation and functioning of microglial cells (83, 84). Microbiota implicated in the pathogenesis of impaired growth in preterm newborns were shown to cause up-regulation of markers of neuroinflammation in germ-free mice (85).
*2) The role of microbiome metabolites on the Gut-Brain Axis:* The gut microbiome produces various metabolites that not only affect the development of the gut immune system, but also affect the Gut-Brain Axis. For example, indigestible oligosaccharides are fermented in the colon, resulting in production of short chain fatty acids (SCFAs) which affect homeostasis of the Gut-Brain Axis (26, 86). This action is partly exerted through modulating tryptophan metabolism (44). Namely, the microbiome in the gut metabolizes tryptophan to tryptamine, indole, and indol-3-proprionic acid (IPA), and all these metabolites exert either activating or inhibitory actions on aryl hydrocarbon receptors (AHRs) (44), subsequently affecting the gut immune system, and the gut-brain axis.
3) *Abnormalities in the gut microbiome and their effects on the brain in ASD*: It has been shown that GI symptoms are one of the most common comorbid conditions, which is partly attributed to dysbiosis, as reviewed elsewhere (87). Changes in the gut microbiome in ASD subjects has been repeatedly shown by multiple authors (82). One study showed that transplanting gut microbiota from ASD patients to the germ-free mice induced ASD like behavioral symptoms (88). Butyrate (BT) is one of the major components of SCFAs. BT is thought to be neuroprotective, partly through suppressing inflammatory responses in the macrophage lineage cells in the gut (26). One study showed that bacterial taxa producing BT are lower in ASD subjects than controls (89). Others also reported that 4-ethylphenylsulfate (4-EPS), a toxic metabolite of the gut microbiota, which has been implicated in the pathogenesis of ASD behavioral symptoms, was high in the MIA model, a rodent model of autism (90). This was normalized by colonization of *Bacteroides Fragilis* (90). SCFAs are also known to affect the metabolism of tryptophan and will further affect the brain when an imbalance of SCFAs develop due to dysbiosis (87). In ASD subjects, reduced biodiversity of the microbiota has been reported which may be associated with SCFAs such as propionic acid in the gut (91, 92). Propionic acid has been implicated in the ASD pathogenesis with creation of the rat model of autism as a propionic acid-induced autism (93). In fact, both propionate and butyrate are known to modify the expression of numerous genes in neuronal cells and will impact their functions (94, 95).
4) *Intestinal Barrier Dysfunction and the Microbiota-Gut-Brain-Axis:* ASD subjects may be more easily affected by the changes in the gut microbiome secondary to their increased gut permeability, allowing increased flux of chemical mediators and even inflammatory cells from the gut (80). Based on findings in the propionic acid-induced animal model of autism, increased gut permeability is attributed to worsening ASD symptoms in certain conditions (96). Previous study also reported increase in the gut permeability at a higher frequency in ASD subjects than neurotypical controls (97).

As summarized above, abnormalities in the gut microbiome have been widely recognized in ASD. However, the treatment options to correct such abnormalities of microbiome have not been extensively tested. The study results may also be affected by marked heterogeneity of ASD subjects. The recent meta-analysis of the use of probiotics revealed overall favorable responses to probiotics in ASD subjects, but these data are generated in the studies with small numbers of subjects (82). It may be promising, but more information is needed to better understand which commensal floras are more beneficial and can aid in controlling ASD behavioral symptoms. The studies addressing effects of prebiotics, synbiotics, and a combination of prebiotics, probiotics, and synbiotics, yielded mixed and inconclusive results by recent meta-analysis (82). Likewise, butyrate, one of SCFAs, is reported to have anti-inflammatory and neuroprotective effects in animal models in BTBR mice (98) and the rat MIA model induced by maternal injection of LPS (99). However, there are no clinical trials of butyrate that have reproduced the favorable effects of butyrate seen in the preclinical data regarding ASD subjects.

The use of fecal microbiota transplant (FMT) for correcting dysbiosis in ASD patients has recently begun. Recent studies of FMT have yielded promising results: Kang, et al. reported improvement of GI symptoms and ASD behavioral symptoms following treatment of FMT for 8 weeks in 80% of participants (100). The same group also reported that in the follow-up of the 18 participants who were responsive to the FMT, improvement of GI and behavioral symptoms was maintained in most of the subjects for 2 years after the completion of the FMT (101). Similar beneficial effects of FMT have also been reported by another group (102). The recent review of meta-analysis reported significant improvement in ABC as well as Child Autism Rating Scale scores following FMT (103), FMT may provide another treatment option in ASD subjects who exhibit evidence of chronic GI inflammation. However, it is also of note that in the published studies, ASD subjects with other notable co-morbid conditions such as seizure disorders have been excluded in these studies. However, in epilepsy, a potential effect of gut microbiome has been suspected (104). Epilepsy has become a major co-morbid condition in older ASD children and young adults suffering from ASD. FMT may eventually be expanded for treating ASD subjects with other serious co-morbid conditions associated with CNS inflammation such as seizures.

## 3 Immunomodulating agents utilized for autoimmune/autoinflammatory and post-infectious encephalopathy – potential applications to ASD subjects

Recently, more and more immunomodulating agents have been used for controlling neuroinflammation associated with autoimmune/autoinflammatory and/or chronic inflammatory conditions. Some of these agents have been used in ASD subjects under diagnosis of autoimmune and autoinflammatory conditions. However, except for corticosteroids and intravenous immunoglobulin (IVIg), most of these agents are not specifically used for targeting ASD subjects. In this section, immunomodulating agents that could be potentially applied for controlling neuroinflammation in ASD subjects will be discussed. The effects of corticosteroids and IVIg will be discussed first, since published studies for treating ASD subjects are available. Then other immunomodulating agents that have been used for controlling neuroinflammation in various conditions, but not specifically used for ASD subjects will be discussed. ASD subjects referred to the author’s clinic have often been treated with these agents by other providers under diagnoses of various conditions. These include autoimmune encephalitis (AE), pediatric acute-onset neuropsychiatric syndrome (PANS), pediatric acute neuropsychiatric disorders associated with Streptococcal infection (PANDAS), and post-infectious inflammation associated with COVID-19, which is commonly referred as to long COVID. Since newly emerging immunomodulating agents exert potent actions with the possibility of even more hazardous side effects than described in the previous section, it will be necessary for clinicians to understand the underlying mechanisms of action and scientific rationale for their use. Nevertheless, it should be noted that these additional treatment options will be welcome to those ASD subjects who have difficulty in responding to the treatment measures described in section 2.

### 3.1 Corticosteroids

Corticosteroids (CS) have been used in numerous autoimmune and inflammatory conditions including conditions described in the previous paragraph. They exerts a wide variety of anti-inflammatory and immunosuppressive actions (105). Although CS provide quick symptomatic relief in autoimmune and inflammatory conditions, they are typically used in the initial stage aiming for inducing remission, secondary to significant side effects (105). CS have also been used for treating various psychiatric disorders associated with neuroinflammation (106). CS play pivotal roles in the stress responses mediated by the hypothalamic pituitary adrenal (HPA) axis. In some ASD subjects, altered or impaired function of the HPA axis has been reported (106, 107). Therefore, in certain conditions, CS may provide some therapeutic effects in ASD subjects.

There are two reports of randomized, placebo-controlled trials of CS on small numbers of ASD subjects. One study (108) tried 1 mg/kg/day prednisolone for 12 weeks as an add-on treatment to risperidone in 37 ASD subjects (single blinded): the authors report improvement of ABC subscale scores (irritability, hyperactivity, lethargy, and stereotypy) (108). Another study (109) used 1 mg/kg/dose prednisolone for 24 weeks and tapering off over 9 weeks (N=20 in the placebo group and N=18 in the trial group); authors report significant improvement of language scores in ASD subjects treated with prednisolone (109). However, in these studies, there was no careful evaluation for components of neuroinflammation in the ASD study subjects. Various case reports or case series reported some benefits of CS. However, CS is also known to cause significant short-term and long-term side effects. One of the concerning side effects of CS in ASD subjects is psychomotor agitation, often manifested as mood swings, especially with a high dose of CS (105). Therefore, CS may be a suitable measure for acute care, controlling the initial stage and/or acute exacerbation of neuroinflammation.

### 3.2 Intravenous immunoglobulin

In addition to providing functional antibodies, IVIg exerts a wide range of immunoregulatory actions, and has been used for treating autoimmune and post-infectious inflammatory conditions involving the CNS and/or peripheral nervous system. Such neurological conditions treated by IVIg include Guillain-Barré syndrome (GBS), chronic inflammatory demyelinating polyneuropathy (CIDP), AE, and PANS/PANDAS.

1) *Effects of IVIg on innate immunity*: A part of the anti-inflammatory effects of IVIg has been attributed to antibodies interacting with the activated form of complement 3 (C3b), blocking downstream cascade of complement activation, thereby inhibiting complement mediated inflammatory processes. This action of IVIg was shown to have prevented neuronal cell death in an animal model of stroke (110). IVIg is also known to inhibit the activation of innate immune cells [macrophage/monocyte lineage cells and dendritic cells (DCs)] through induction of Fas-mediated apoptosis and triggering the production of counter-regulatory cytokines. In addition, IVIg contains neutralizing antibodies that bind to cytokines produced by innate immune cells (111). It also modifies both activating and inhibitory signaling through Fcγ receptors (111). Such effects of IVIg on Fcγ receptors are attributed to α2,6-sialic acid portion of immunoglobulins that exert actions via the DC-SIGN signaling in murine models (112, 113). However, it is unclear whether the same actions take place in human cells. IVIg has also been shown to suppress B cell activation/proliferation by agonistic binding to inhibitory cell surface receptors on B cells (111). Antibodies contained in IVIg have also been shown to directly neutralize pathogenic autoantibodies (111).
2) *Effects of IVIg on adaptive immunity*: IVIg has been shown to exert a regulatory effect on the balance on Treg and inflammatory-prone, type 17 T-helper (Th17) cell differentiation and function. In Kawasaki disease (KD) for which IVIg serves as the 1^st^ line treatment measure, IVIg promotes Treg cell function/differentiation and suppresses the production of inflammatory cytokines by Th17 cells (114, 115). Effects of IVIg on the balance of Th1/Th17 and Treg cells has been reported in GBS and CIDP patients, as well (116, 117).

IVIg is reported to exert actions on many other immune cells, including neutrophils and NK cells. However, the actions of IVIg may vary depending on donor pool and how the product was prepared. This makes it difficult to assess the effects of IVIg on ASD subjects, whose degrees of neuroinflammation may also vary. Previous studies assessing the effects of IVIg on ASD children reveled mixed results (118, 119). In one double-blind, placebo-controlled crossover study, 12 ASD subjects without known immunodeficiency were given a single dose of IVIg. Authors report no improvement of clinical ratings as compared to controls treated with placebo (120). On the other hand, in 31 ASD children treated under diagnosis of AE, authors reported modest, but statistically significant improvement in subscale scores of ABC and SRS (social responsiveness scores) (121). In this study, AE diagnosis appears not based on the standard measures for seropositive AE. Instead, ASD subjects were recommended to have IVIg treatment, based on positive non-specific inflammatory markers. These results indicates that IVIg may be a feasible option for ASD subjects with clear evidence of antibody deficiency and/or evidence of immune-dysregulatory conditions that have been shown to benefit from the immunoregulatory actions of IVIg.

### 3.3 B cell targeted therapy

1) *Rituximab (B cell ablation therapy):* AE is a rare autoimmune condition affecting the CNS through the production on autoantibodies that attack neuronal cells (seropositive AE) and/or through cell mediated immunity (seronegative AE). AE is rare in all age groups and clinical presentation may vary markedly. In addition, presenting symptoms may overlap ASD behavioral symptoms. AE was initially reported in patients with antibodies against the NMDA receptor, which remains the leading cause of AE (122). Onset of AE symptoms may be rapid. However, in some cases, AE symptoms can be subtle and may have symptoms such as developmental regression, resembling developmental issues often seen in ASD (123). AE diagnosis requires extensive neuroimaging, analysis of cerebrospinal fluid (CSF) including autoantibodies associated with AE, and electroencephalogram (EEG) to support AE diagnosis. The possibility of misdiagnosing someone with AE with ASD exists, since there may be common underlying mechanisms associated with both AE and ASD (123). In addition to the agents commonly used to control the acute stage (steroid, plasmapheresis, and IVIg), other immunomodulating agents have been utilized to achieve long-term remission in AE. Rituximab has been frequently used as the 2^nd^ line treatment measure for this purpose (124).
Rituximab is a chimeric monoclonal antibody targeting CD20 which is expressed predominantly on mature B cells. The therapeutic effects of rituximab are attributed to the deletion of antibody producing memory B cells, and also prevention on generation of new plasma cells (125). Long lived mature plasma cells which does not express CD20 will keep producing autoantibodies, so that autoantibody production, albeit low in degree, is expected to continue in AE patients (125, 126). Repopulation of deleted B lineage cells is reported to start to occur 26 weeks after the initial treatment (127). Thus, rituximab is typically given every 6 months in AE patients (127). Rituximab has also been used for treating refractory patients with PANDAS/PANS in select cases (128, 129). However, no double blinded, placebo-controlled trials have been reported in AE or PANS/PANDAS subjects. In the author’s experience, there are ASD subjects treated with rituximab under diagnosis of AE, PANS, or PANDAS by other providers, but their responses appear to be mixed. Rituximab use may be limited to ASD patients for whom there is clear-cut evidence that autoantibodies or autoreactive B cells play a role in their comorbid medical conditions.
2) *Tocilizumab*: In AE patients without detectable autoantibodies (seronegative AE) or refractory to B cell targeted treatments, other immunomodulating agents that target other pathways have been tried. Blockers targeting IL-6 signaling have been used for treating AE, especially seronegative AE (130). Tocilizumab is a humanized monoclonal antibody (mAb) that targets soluble, and membrane attached IL-6 receptors (IL-6R) (130, 131). It blocks the function of IL-6, a pleotropic cytokine, that affects not only B cell differentiation, but also affects the function of T cells and innate immune cells (131). In a cohort study conducted at a single institution, tocilizumab was reported to have favorable effects in patients diagnosed with AE and refractory to rituximab (132). The same group reported better outcomes (better modified ranking scales) in the escalation treatment using tocilizumab, compared to controls: 60 out of 80 AE patients that were included in this study were categorized as seronegative AE (133). It is unclear whether any reported studies included ASD subjects. Currently, no published data exists regarding trials of tocilizumab in ASD subjects diagnosed with AE or other autoimmune conditions. Our clinic has experienced some ASD subjects treated with tocilizumab elsewhere, but their responses appear to be mixed. It is unclear at this time which ASD subjects, if any, would benefit from tocilizumab, in the absence of good biomarkers.

### 3.4 Biologics and blockers of inflammasome signaling pathway

As blockers of inflammasome, colchicine and anakinra, IL-1ß blocker, have been extensively used for controlling autoinflammatory syndromes caused by pathogenic gene variants that render dysregulation inflammasome activation (134, 135). Colchicine and anakinra have also been used for treating multiple autoimmune and autoinflammatory conditions. Activation of innate immunity is often associated with activation of inflammasome signaling pathways and blockers of inflammasome may thus provide additional therapeutic options for ASD subjects who have evidence of innate immune abnormalities. Therapeutic utility of inflammasome blockers (colchicine and anakinra) have also been illustrated in patients with long COVID (136–139), since activation of type 1 IFN signaling pathway by sars-cov-2 leads to inflammasome activation (140). These agents are readily applicable for treating ASD subjects suffering from long COVID. Indeed, the author also observed favorable effects of colchicine and anakinra in ASD subjects suffering from long COVID in the author’s clinic. The therapeutic actions of colchicine and anakinra are summarized below:

1) *Colchicine – an old medication for autoinflammatory conditions*: Colchicine is known to affect the actions of tubulins, which play key roles in chemotaxis and phagocytosis of innate immune cells (136). It also inhibits NLRP3, and subsequently blocks IL-1ß induced inflammasome activation and production of TNF-α and IL-6 (141, 142). Colchicine’s action on neutrophils also inhibits neutrophil-platelet interactions, preventing thrombosis triggered by neutrophilic inflammation (142, 143). Given these actions, colchicine, an established medication commonly used for autoimmune/autoinflammatory conditions, is expected to be useful for controlling neutrophilic inflammation triggered by Th17 cells and/or innate immune cells. Since colchicine is a strong inhibitor of P450 3A4, it is necessary to evaluate all drugs that patients are already on for possible drug-interactions. Nevertheless, the above-described actions of colchicine appear promising for controlling COVID-19 induced neuroinflammation. Indeed, several studies reported decreased mortality in severe COVID-19 cases with the use of colchicine (136, 144). However, the current published reports of colchicine focus on its effects on cardiovascular conditions associated with long COVID, and little information is available regarding its actions on neuropsychiatric symptoms. The author has experienced favorable effects with the use of colchicine in ASD subjects suffering from long COVID, with improvement in behavioral symptoms shown by the ABC (145). However, at this time, there is no published data of a trial of colchicine in ASD subjects, with or without long COVID.
2) *Anakinra*: Anakinra, a soluble IL-1 receptor antagonist (IL-1Ra), is a recombinant product of human IL-1ra. It has been used for treating various autoimmune and autoinflammatory conditions (146). Following the COVID-19 pandemic, IL-1ß has been shown to be a key cytokine, causing cytokine storm and subsequent hyper-immune activation in severe COVID-19 cases (137, 139). These findings indicate that there is utility of anakinra for treating long COVID. One study reported marked increase in spontaneous production of IL-1ß from NLRP3 inflammasome in severe COVID-19 cases and subsequent favorable responses to anakinra (138). IL-1ß is also implicated in the pathogenesis of epilepsy associated with neuroinflammation and this prompted the use of anakinra for treatment of refractory seizure disorders, since anakinra is a small molecule that can pass through the intact blood brain barrier (BBB) (147, 148). The author has experienced favorable effects with anakinra in patients with refractory seizure disorder as an adjunctive treatment (75). IL-1ß has also been implicated in the pathogenesis of MIA, as described in the Introduction section. In animal models of chorioamnionitis induced by Group B streptococcal infection, neurobehavioral impairment was attenuated by an IL-1ß blockade by anakinra (149). Anakinra may be a reasonable therapeutic option for ASD subjects suffering from refractory seizures, long COVID, and autoinflammatory conditions refractory to the 1^st^ intervention measures.

### 3.5 Blockers of mTOR pathways

As described earlier, candidate genes implicated in the pathogenesis of ASD include those associated with PI-3K/Akt/mTOR signaling pathway (65, 66). Importance of this signaling pathway was illustrated in patients with TS, and mTOR inhibitors have been used for controlling refractory seizures in TS patients (67). Given the proposed roles of the PI-3K/Akt/mTOR signaling pathway in ASD pathogenesis, mTOR inhibitors may also have favorable effects on neurodevelopment or cognitive functioning. In the rodent model of haplo-insufficient TS complex (TSC), everolimus, a mTOR inhibitor that was developed for TS seizure control, was reported to have attenuated impairment of social deficits (150). Such favorable effects of mTOR inhibitors were also shown in the rodent model of ASD created by silencing the *Cntnap2* gene, thereby causing hyperactivation of the Akt-mTOR signaling (151).

Hyperactivation of PI-3K/Akt/mTOR signaling pathway has also been reported in patients with COVID-19. Blockers of this signaling pathway has also been proposed as possible therapeutic options for severe COVID-19 (152). It was proposed that mTOR inhibitor can exert favorable therapeutic effects on severe COVID-19 patients by augmenting autophagy along with inhibiting viral replication (153, 154). However, at this time, there is no clinical trials of mTOR inhibitors in ASD subjects without identified mutations of PI-3K/Akt/mTOR pathway. In ASD subjects with clear evidence of hyperactivation of this signaling pathway and/or in those with long COVID refractory to other measures, inhibitors targeting this pathway may provide an additional treatment option (155, 156). Likewise, there are no data of a trial of mTOR inhibitors in ASD subjects without pathogenic mutations of PI-3K/Akt/mTOR pathways. ASD with treatment resistant seizures may benefit from the use of mTOR inhibitors.

### 3.6 Blockers of type 1 IFN signaling and downstream signaling

As summarized in the introduction section, epigenetic changes in innate immunity can cause prolonged effects, which are now referred as to IIM (8, 157). Previous studies reported by the author and her colleagues revealed evidence of on-going innate immune abnormalities associated with altered IIM in some ASD subjects (158–160). One of the innate immune pathways triggered by various viruses including sars-cov-2 is the type 1 IFN signaling pathways. Induction of dysregulated IIM by SARS-CoV-2 may partly explain the long term sequelae of long COVID (161).

Patients with primary immunodeficiency caused by excessive production of type 1 IFNs called as interferonopathies, present with autoinflammatory and subsequent autoimmune conditions (162). These patients frequently present with neuropsychiatric symptoms. Long COVID patients often reveal neuropsychiatric symptoms and neurological deficits (163, 164). In animal models of long COVID, persistent neuroinflammation has been shown to affect multiple neuronal cells, including microglial cells which appear to play a crucial role in COVID induced persistent neuroinflammation (164, 165). AE like symptoms associated with COVID-19 have also been reported (166, 167). In these patients described above, neuroinflammation is expected to be better managed by immunomodulating agents that target signaling pathways activated by type 1 IFNs. It is also of note that type 1 signaling pathways and resultant Th17 cell activation have been implicated in the pathogenesis of various autoimmune conditions as seen in patients diagnosed with interferonopathies (162). Key down-stream signaling molecules in this pathway are Janus kinases (JAK). In fact, JAK inhibitors have emerged for controlling the above described autoimmune/autoinflammatory conditions triggered by type 1 IFN signaling (168, 169). In this section, JAK inhibitors and Th17 cell targeted treatment measures will be discussed.

1) *JAK inhibitors*: Janus kinases (JAKs) act as signal transducers and 4 mammalian members are identified: JAK1, JAK2, JAK3, and TYK2 (170, 171). All JAKs, except for JAK3, which is expressed only in hematopoietic and lymphoid cells, are expressed ubiquitously, and play a crucial role in the JAK-STAT (signal transducers and activators of the transcription) pathways (171). The JAK-STAT pathway transduces signaling from multiple cytokine receptors. JAK3 mediates signals from type 1 cytokines and thus deficiency of JAK3 lead to severe combined immunodeficiency (SCID) (172). On the other hand, dysregulated activation of the JAK-STAT pathway will lead to chronic inflammatory conditions, implicated in autoimmune, allergic, and autoinflammatory conditions (173). In patients with interferonopathies, type 1 IFN signaling involving JAK1/JAK2 may be therapeutic targets for these patients (162). Likewise, cytokines noted to be up-regulated in patients with severe COVID-19 utilize the JAK/STAT pathway and levels of these cytokines are reported to be positively associated with the disease outcome/mortality of COVID-19 (174). Therefore, JAK inhibitors have been used for treating COVID-19 as well (170).
Baricitinib, a JAK1/JAK2 inhibitor, has been used for treating severe COVID-19 cases requiring hospitalization. In a randomized, double-blind, placebo-controlled trial for severe COVID-19 patients, baricitinib was reported to have caused less adverse reactions when used as an adjunctive therapy to remdesivir and dexamethasone (175). Multiple open-label studies have also reported beneficial effects of baricitinib for treating severe COVID-19 (176). Similar beneficial effects of tofacitinib, a JAK3/1 inhibitor, was reported in severe COVID-19 cases (177, 178). Beneficial effects of ruxolitinib, a JAK1/2 inhibitor, have also been reported in severe COVID-19 cases (179). With the increase in reports of the favorable effects of baricitinib, FDA issued the EUA (emergency use authorization) for its use as an adjunct treatment with remdesivir for the treatment of hospitalized COVID-19 patients older than 2 years of age. Newly available JAK1/2 inhibitors (upadacitinib and abrocitinib) may even be more effective for controlling type 1 IFN induced immune activation (180, 181).
There are no reports of clinical trials of JAK inhibitors in ASD patients. In ASD subjects with pre-existing immunodysregulatory conditions involving the JAK/STAT pathways, JAK inhibitor may provide an additional treatment option for controlling neuropsychiatric symptoms, especially in those suffering from long COVID. Oral intake and the acceptable safety profiles of JAK inhibitors may make it an easier option for ASD subjects (168, 169).
2) *Th17 cell targeted therapy*: Type 17 T-helper (Th17) cells differentiate from pluripotent T cells through induction of Th17 specific transcription factor RORγt (RAR related orphan receptor-γt) which signals through the STAT3/JAK3 pathway, rendering Th17 cell differentiation in the presence of IL-1ß, IL-6 and TGF-ß (182). RORγt augments expression of IL-17 and IL-23 receptors and IL-23 produced by innate immune cells binds to IL-23R, further augmenting induction of RORγt and IL-17 (183). Although IL-23 does not induce Th17 differentiation, it stabilizes Th17 cells. Th17 cells are known to exert a major defense against fungal pathogens through activation of neutrophils, but dysregulation of IL-23/IL-17 axis has been implicated in the pathogenesis of various autoimmune and inflammatory conditions (184, 185). Immunomodulating agents targeting the IL-17/IL-23 axis have been successfully used for treating multiple autoimmune conditions (185).
After the onset of the COVID-19 pandemic, marked activation of the IL-17/IL-23 axis following COVID-19 has become also apparent (186–189). This may also be the results of an imbalance in regulatory T (Treg) and Th17 cells during the acute stage of COVID-19 as well as in long COVID (190). Restoring the Treg/Th17 balance with the use of a Treg cell inducing cytokine (IL-2) or IL-17 inhibitors may be a possible treatment option for long COVID patients (190). There are report of increase in serum levels of IL-17 along with IL-2 in long COVID patients (191). JAK inhibitors inhibit STAT mediated signaling of IL-17 inducing cytokines, as well as Th17 produced cytokines. Therefore, JAK inhibitors may also exert beneficial effects by suppressing Th17 differentiation/functions in ASD subjects suffering from autoimmune conditions or chronic inflammatory conditions like long COVID. Immunomodulating agents targeting Th17/IL-23 axis have been developed for controlling IBD and skin inflammatory conditions like plaque psoriasis (192, 193). ASD subjects are known to suffer from chronic GI symptoms (16). In addition, skin pruritus/discomfort are expected to cause worsening behavioral symptoms (194). ASD subjects suffering from the above-described medical conditions are likely to benefit from medications targeting IL-17/IL-23 axis. There are no reports of Th17 targeted therapies tried specifically in ASD subjects. However, the author experienced that several ASD subjects treated with Th17/IL-23 targeted therapies (ustekinumab, secukinumab, and risankizumab) for co-morbid conditions, as described above, revealed beneficial effects on their ASD behavioral symptoms. ASD subjects suffering from long COVID may also benefit from these medications. However, it will be necessary to carefully select ASD subjects for a trial using inhibitors of the IL-17/IL-23 axis.

## 4 Conclusions

In this review, the author discussed various immunomodulating agents as possible treatments for neuroinflammation in ASD subjects based on solid scientific rationale and clinical trial data that were available at the time of preparation of this manuscript. In addition, this review also discussed the potential use of emerging immunomodulating agents including biologics. These agents were developed in recent years and many new agents are now in the pipeline. These agents may provide additional treatment options for ASD subjects. However, published data of clinical trials in ASD subjects are scant for emerging biologics and other immunomodulating agents. Therefore, discussion regarding scientific rationale for biologics for this review was based on pre-clinical studies (animal models), and results of studies from other medical conditions. It cannot be emphasized enough that ASD subjects are markedly heterogenous, and therefore, careful evaluation must take place for assessing treatment options of immunomodulating agents based on clinical and laboratory findings. In addition, it is imperative for medical providers to have a good understanding of the mechanisms of actions for these agents in order to provide optimal treatment measures safely.

## Author contributions

HJ: Conceptualization, Funding acquisition, Validation, Writing – original draft, Writing – review & editing.
